# Analysis of Imprinted Gene Expression in Normal Fertilized and Uniparental Preimplantation Porcine Embryos

**DOI:** 10.1371/journal.pone.0022216

**Published:** 2011-07-15

**Authors:** Chi-Hun Park, Kyung-Jun Uh, Brendan P. Mulligan, Eui-Bae Jeung, Sang-Hwan Hyun, Taeyoung Shin, Hakhyun Ka, Chang-Kyu Lee

**Affiliations:** 1 Sooam Biotech Research Foundation, Seoul, Republic of Korea; 2 National Institute of Animal Science, RDA, Suwon, Republic of Korea; 3 College of Veterinary Medicine, Chungbuk National University, Cheongju, Chungbuk, Republic of Korea; 4 Division of Biological Science and Technology, and Institute of Biomaterials, Yonsei University, Wonju, Republic of Korea; 5 Department of Agricultural Biotechnology, Research Institute for Agriculture and Life Science, Seoul National University, Seoul, Republic of Korea; The Babraham Institute, United Kingdom

## Abstract

In the present study quantitative real-time PCR was used to determine the expression status of eight imprinted genes (*GRB10*, *H19*, *IGF2R*, *XIST*, *IGF2*, *NNAT*, *PEG1* and *PEG10*) during preimplantation development, in normal fertilized and uniparental porcine embryos. The results demonstrated that, in all observed embryo samples, a non imprinted gene expression pattern up to the 16-cell stage of development was common for most genes. This was true for all classes of embryo, regardless of parental-origins and the direction of imprint. However, several differentially expressed genes (*H19*, *IGF2*, *XIST* and *PEG10*) were detected amongst the classes at the blastocyst stage of development. Most interestingly and despite the fact that maternally and paternally expressed genes should not be expressed in androgenones and parthenogenones, respectively, both uniparental embryos expressed these genes when tested for in this study. In order to account for this phenomenon, we compared the expression patterns of eight imprinted genes along with the methylation status of the *IGF2/H19* DMR3 in haploid and diploid parthenogenetic embryos. Our findings revealed that *IGF2*, *NNAT* and *PEG10* were silenced in haploid but not diploid parthenogenetic blastocysts and differential methylation of the *IGF2/H19* DMR3 was consistently observed between haploid and diploid parthenogenetic blastocysts. These results appear to suggest that there exists a process to adjust the expression status of imprinted genes in diploid parthenogenetic embryos and that this phenomenon may be associated with altered methylation at an imprinting control region. In addition we believe that imprinted expression occurs in at least four genes, namely *H19*, *IGF2*, *XIST* and *PEG10* in porcine blastocyst stage embryos.

## Introduction

Uniparental mouse embryos consisting entirely of either a paternally or maternally inherited genome can develop through early preimplantation stages, but are growth retarded at embryonic day 10 [1]. There are distinctions between the phenotypic features of the maternal and paternal genomes; the maternal genome is likely to be critical for the development of the embryo proper, whereas the paternal one is necessary for the development of the extraembryonic tissues. These differential functions of the parental alleles in development are largely associated with imprinting mechanisms, which lead to the selective expression of certain loci according to their parental origin [2].

Since it has been demonstrated that many imprinted genes play an important role in normal fetal and placental development, imprinting mechanisms in pre- and post-implantation development have been studied for a number of species. It has been clearly demonstrated that parental-specific methylation imprint marks are established during gametogenesis and maintained throughout development [3]. It has been suggested that assisted reproductive technology (ART) procedures affect the imprinting states of preimplantation embryos. Environmental factors such as culture conditions and manipulations may influence methylation patterns and thus affect the expression of imprinted genes in embryos at various developmental stages [4], [5]. Moreover, human embryos produced via *in vitro* fertilization (IVF) or intracytoplasmic sperm injection (ICSI), show increased incidences of imprinting-related disorders such as Beckwith-Wiedemann syndrome [6]. It has also been demonstrated that imprinting errors due to aberrant reprogramming in cloned embryos directly influence development. For example, it is known that aberrant *IGF2R* expression in preimplantation embryos is associated with large offspring syndrome [7]. As such, many imprinted genes have been considered as valuable genetic markers for evaluating the developmental ability and normality of *in vitro* produced embryos and their derivatives, embryonic stem cells.

In pigs, a few imprinted genes have been found to be expressed monoallelically in somatic tissues [8], [9]. Recently, we and others have confirmed the methylation patterns of *IGF2/H19* DMRs in preimplantation embryos and in primordial germ cells, respectively [10], [11]. However, detailed knowledge about epigenetic imprints at early stages of embryogenesis remains largely absent in this species.

In this study, in order to analyze allele-specific expression patterns of imprinted genes in porcine preimplantation embryos, eight genes, including both paternally (*IGF2*, *NNAT*, *PEG1* and *PEG10*) and maternally (*GRB10*, *H19*, *IGF2R* and *XIST*) expressed genes, governing fetal and placental growth, were selected. Amongst the paternally expressed genes, insulin-like growth factor 2 (*Igf2r*) was the first imprinted gene to be identified in mammals and plays a crucial role in fetal growth and placental function [12]. The neuronatin (NNAT) protein functions as a regulator of ion channels during brain development and is also involved in insulin secretion in pancreatic β-cells [13]. The porcine *PEG1* gene (a.k.a. *MEST*) is known to be imprinted in fetal tissues and the placenta [9]. As an imprinted gene acquired from a retrotransposon, *Peg10*, is known to play an essential role in the placental development of mice [14]. Further to this it has recently been shown that the *PEG10* gene is monoallelically expressed in somatic tissues in pigs [15].

*GRB10*, *H19*, *IGF2R*, and *XIST* are known to be maternally expressed genes. Growth factor receptor-bound protein 10 (GRB10), which is an adaptor protein, is capable of binding to receptor tyrosine kinases. This gene acts as a potent growth inhibitor during the fetal and placental development of mice [16]. The *H19* gene is imprinted in an opposite manner to its neighboring *Igf2* gene and produces a developmentally regulated transcript that is mRNA-like noncoding RNA [17]. *Igf2r* encodes a multifunctional receptor that is involved in the regulations of cell growth and differentiation. Knockout experiments have demonstrated that *Igf2r*-null mice exhibit fetal overgrowth or late gestational lethality [18]. The mouse *Xist* gene, which is believed to govern the X-chromosome inactivation (XCI) process, is expressed exclusively from one of two X chromosomes in which transcriptional silencing occurs. XCI is thought to be a critical process necessary to achieve equivalent levels of X-linked gene expression between males (XY) and females (XX) [19].

In order to determine the allele-specific expression status in the genome of a normal diploid embryo, suitable polymorphic markers are required to distinguish between maternal and paternal alleles. In this regard, the laboratory mouse is the most convenient model system as a wealth of different genotypes exists between inbred strains and a great deal is known about the genetics of mice in general [20]. In other species, however, it is much more difficult to identify key genetic markers; as there is usually an absence of such readily available inbred animal lines. Considering these limitations when working with a non mouse model, uniparental embryos provide an effective model system for studies on genomic imprinting [1]. To achieve this, we produced three different types of porcine embryo, *in vitro* fertilized (IVF), parthenogenetic (PG) and androgenetic (AG) embryos. The developmental potential of these embryos along with imprinted gene expression levels was observed throughout preimplantation development. Furthermore the methylation pattern of the *IGF2/H19* differentially methylated region 3 (DMR3) was determined in blastocyst stage embryos of parthenogenetic origin. Our results demonstrate that several imprinted genes exhibit differential expression patterns amongst embryo types specific to parental origins. For some genes, improper expression in uniparental blastocysts was associated with an altered methylation status, suggesting that there may be a gene dosage compensation mechanism or loss of imprinting in diploid uniparental embryos.

## Materials and Methods

Unless otherwise stated, all chemicals were obtained from Sigma-Aldrich Corp. (St. Louis, MO). This study was conducted in accordance with the *Guide for the Care and Use of Agricultural Animals in Agricultural Research and Teaching*, published by the Federation of Animal Science Societies, 1st revised ed., 1999.

### Production of Porcine Embryos

#### In vitro maturation (IVM)

The ovaries used were collected from pre-pubertal gilts at a local slaughter house and transported to the laboratory within 1 h at 37°C. Only cumulus-oocyte complexes (COCs) were obtained from follicles 3–6 mm in diameter using 18-gauge micro needles. The follicular contents were pooled in a 50 ml conical tube and then allowed to sediment after which the supernatant was carefully discarded. The sediment was washed once with TL-Hepes-PVA medium (Tyrode's lactate-Hepes medium supplemented with 0.01% polyvinyl alcohol). Oocytes possessing an evenly granulated cytoplasm and a compact surrounding cumulus mass were collected, and washed twice with TL-Hepes-PVA medium. After washing, 40–50 COCs were transferred to 500 µl of an IVM medium (TCM-199; Life Technologies, Rockville, MD), supplemented with 10 ng/ml epidermal growth factor (EGF), 4 IU/ml eCG (Intervet, Boxmeer, The Netherlands), hCG (Intervet) and 10% (v/v) porcine follicular fluid (pFF) and were cultured for 22 h. After 22 h of culture, the COCs were transferred to an IVM medium without hormones and were cultured for a further 22 h at 39°C in an atmosphere containing 5% CO_2_ and 100% humidity.

For the production of *in vitro* embryos by *in vitro* fertilization, parthenogenesis, and androgenesis, the COCs were treated with 0.1% hyaluronidase in IVM medium to remove the cumulus cells.

#### In vitro fertilization (IVF)

Briefly, 15–20 oocytes were placed into 40 µl drops of modified Tris-buffered medium (mTBM) that had been covered with warm mineral oil in a 35 mm dish. Frozen semen was thawed by incubation at 39°C for 60 seconds and was washed twice by centrifugation at 350× *g* for 3 min in PBS. The sperm pellet was then resuspended and adjusted to the concentration of 2×10^6^ sperm/ml. The appropriate concentration of sperm was introduced into the oocyte containing medium drop and these cells were then incubated for 6 h at 39°C. After fertilization, excess spermatozoa were removed from oocytes by a repetitive pipetting action, and fertilized oocytes were then washed three times in a culture medium (NCSU-23) [21] containing 2% EAA; MEM essential amino acid solution, 1% NEAA; MEM nonessential amino acid solution and 50 µM β-mercaptoethanol.

#### Parthenogenesis

Diploid and haploid parthenogenetic embryos were generated via the electrical activation method with or without cytochalasin D treatment to suppress the extrusion of the second polar body. Briefly, cumulus-free oocytes were washed twice in a 280 mM mannitol solution containing 0.5 mM Hepes, 0.1 mM CaCl_2_ and 0.1 mM MgCl_2_. These treated oocytes were then placed in an electrode-chamber and activated with a single DC pulse (2.0 kV/cm 30 µs) using a BTX Electro-cell Manipulator (BTX, CA, USA). The activated oocytes were cultured in NCSU23 with 7.5 mg/ml cytochalasin D for 1 h. Under these experimental conditions, a greater proportion of oocytes containing one diploid nucleus were obtained with fewer numbers possessing two haploid pronuclei.

#### Androgenesis

As previously stated, androgenetic embryos were produced by the *in vitro* fertilization of enucleated oocytes [22]. Briefly, matured oocytes were enucleated by a squeezing enucleation method that was confirmed using Hoechst 33342 dye under a UV light. The successfully enucleated oocytes were fertilized using the same process as described above, however a sperm fraction (10 µl), with a final concentration of 4×10^6^ sperm/ml, was added for insemination and then co-incubated for 6 h.

#### In Vitro Culture (IVC)

About 30–40 fertilized or electrically activated oocytes were cultured in 4-well dishes containing 500 µl of the same medium. Those zygotes showing two pronuclei (IVF and AG) or one large pronucleus (PG) were selected using Hoechst. 33342 staining 12 to 15 h after fertilization and were then cultured *in vitro* for 168 h. Embryo culture conditions were maintained at 39°C in an atmosphere containing 5% CO_2_, 5% O_2_ and 100% humidity for all embryo cultures. Oocytes and embryos (from the two-cell to blastocyst stage) with good morphological features were selected for experiments and the zona pellucida was removed using 0.5% actinase prior to use. The mean total cell number for blastocysts cultured at Day 7 was counted by staining with Hoechst 33342.

#### Recovery of in vivo blastocysts

Briefly, pubertal gilts displaying estrus were mated with a mature boar. Seven days later, they were slaughtered at a local abattoir, and their reproductive tracts were excised. Blastocysts were recovered following flushing of the uteri twice with 50 ml of PBS containing 1% BSA. Within 30 minutes, mRNA was directly isolated from recovered blastocysts and used for the synthesis of cDNA.

### mRNA synthesis and linear amplification of cDNA

Messenger RNA from pools of 10 oocytes, pools of 3–5 cleavage-stage embryos, and the individual blastocysts was extracted using the Dynabeads mRNA Direct Kit (Dynal Asa, Oslo, Norway) according to the manufacturers' instruction. For cDNA synthesis, the enzyme used was Moloney murine leukemia virus reverse transcriptase (MMLV-RT; Promega, Madison, WI, USA) Using a final volume of 20 µl containing 0.5 mg oligo-dT, RT buffer (1 µl), 10 mM dithiothreitol, 10 mM dNTP, and 10 units of reverse transcription was carried out at 37.5°C for 50 min, and samples were subsequently incubated at 70°C for 15 min to inactivate reverse transcriptase.

For identifying the sex of embryos, linear amplification was carried out with SMART technology (Clontech, Palo Alto, CA, USA) according to the manufacturer's instructions. Briefly, 5 µl of the cDNA was mixed with 45 µl of a master mix (37 µl dH2O, 5 µl 10× Advantage 2 PCR buffer, 1 µl 5′ PCR Primer IIA (10 µM), 1 µl 50× dNTP (10 mM) and 1 µl 50× Advantage 2 Polymerase Mix). PCR was performed as follows, 1 cycle of 94°C for 5 min; 25 cycles of 94°C for 30 sec/65°C for 30 sec, 68°C for 6 min and cooled to 4°C. The amplicons were purified with QIAquick PCR Purification Kit (Qiagen, Valencia CA) and cDNA was eluted in 50 µl of dH_2_O. The cDNA products were eventually identified by detecting SRY gene expression (5′-CGTGAAACTAGAGGAAGTGG-3′ and 3′-ATAGCCCGGGTATTTATCTC-5′ for porcine *SRY*; NM_214452).

### Quantitative Real-Time Polymerase Chain Reaction (qRT-PCR)

To minimize the effect of variability of individual sample quality, amplification yield for each sample was primarily analyzed using qRT-PCR with three house-keeping genes. The following primer sets were used: 5′-GGCCATCACATCGTAGCCCTC-3′ and 3′-TTTTATATCGCCCGTTGACTGGT-5′ for *HPRT*; 5′-GATGCTGGTGCTACGTATGTTGTG-3′ and 3′-AGAAGGGGCAGAGATGACC-5′ for *GAPDH*; the primer information for *β-ACTIN* is displayed in Table 1. Prior to use for experiment cDNA samples with a similar threshold cycle value were frozen. Of these genes, the *β-ACTIN* gene showed by far the most stable expression pattern throughout the preimplantation development from oocyte to blastocyst. This gene was therefore used as an internal control for normalization in this study. The primers for the eight imprinted genes and the *β-ACTIN* gene as an internal control were designed, and then analyzed using quantitative real-time PCR analysis.

**Table 1:** Primer sequences for qRT-PCR.

Gene	Primer sequence 5′-3′	Gene Access no.	Length (bp)
*GRB10*	F:GAGGACCAGCAGTTTAGGA	CV875876	147
	R:GACTTTAACATCCTGCTTGG		
*H19*	F:CTCAAACGACAAGAGATGGT	AY044827	122
	R:AGTGTAGTGGCTCCAGAATG		
*IGF2R*	F:AGGTCTCACCTCTTCAGGTT	AF342812	120
	R:CTGTGCAAATTAAGGCTTCT		
*XIST*	F:ATTCCTGAGGTTTGGGTACT	AJ429140	139
	R:AGTGCAGTTGCCAAATTAT		
*IGF2*	F: AAGAGTGCTCTTCCGTAG	NM_213883	156
	R:TGTCATAGCGGAAGAACTTG		
*NNAT*	F:CGACAATACCAGATTCCTTC	DQ666422	138
	R:CTTGGTCCAGATCAGAATGT		
*PEG1*	F:TCTGAGCTGGAAAGAGTAGC	CO868664	134
	R:GGTGGACTTTGTGAGAGAG		
*PEG10*	F:GTTGTTAATGGCTGGAAGAG	DQ323403	148
	R:AGTCACTTCCCCTTCCTAAG		
*β* ACTIN	F:GTGGACATCAGGAAGGACCTCTA	U07786	137
	R:ATGATCTTGATCTTCATGGTGCT		

Amplification and detection were carried out with the ABI 7300 Real-Time PCR system (Applied Biosystems, Foster City, CA) using a quantitative real-time PCR kit (DyNAmo HS SYBR Green qPCR Kit, Finnzymes, Finland) under the following conditions: 95°C for 15 min, 40 cycles of denaturation at 95°C for 15 s, annealing at 60°C for 30 s, and extension at 72°C for 30 s. The PCR reaction mixture (20 µl) consisted of 100 pmol of forward and reverse primers and 1 µl of cDNA. Results for each sample were collected at least three times. All the threshold cycle (CT) values of imprinted genes were normalized relative to that of the *β-ACTIN* gene, and relative expression ratios were calculated via the 2^−ΔΔ^ Ct method [23]. After qRT PCR, all tested gene amplicons were of the expected sizes, and their specificity was confirmed via sequencing analysis.

### DNA isolation and bisulfite treatment

To estimate the methylation status of the *IGF2/H19* Differentially Methylated Region (DMR) 3, genomic DNA from pools of 100 haploid, 50 diploid PG blastocysts, and 50 IVF blastocysts was isolated. The isolation of genomic DNA from porcine samples was carried out using a commercial spin column (G-spin Genomic DNA extraction kit for Cell/Tissue, iNtRON, Korea), with an additional 6 M Urea (Amresco, USA) and 100 mM dithiothreitol (DTT; Sigma, USA) supplemented in a lysis buffer. The genomic DNA was digested with EcoRI (New England Biolabs, Germany). The Bisulfite treatment of DNA was performed as described in our previous study [11]. Briefly, 200 ng of denatured DNA was sulfonated with 5 M sodium bisulfite (pH 5.0; Sigma) in a thermo-cycler programmed for 6 cycles (3 min at 94°C and 3 hr at 60°C). The bisulfite-treated DNA was purified using the Wizard DNA Clean-Up system (Promega, USA) and desulfonated in 0.3 M NaOH for 25 min at 37°C. The DNA was purified again and then resuspended in distilled water. Subsequently, 5 µl of the aliquot was eventually used as a template for PCR.

### PCR amplification and bisulfite genomic sequencing analysis

Nested PCR amplifications of bisulfite-treated DNA was carried out using the following primers, 5′-GGTTTTAGGGGGATATTTTTT-3′ and 3′-TTAAAAAAACATTACTTCCATATA C-5′ for the outside sets of *IGF2/H19* DMR3, 5′-GATTTTTAGGTTTGTTATTATTT-3′ and 3′-CAAATATTCAATAAAAAAACCC-5′ for the inside sets of *IGF2/H19* DMR3. The PCR amplification was performed with a 2× PCR master mix solution (iNtRON, Korea) containing 0.5 pmol of the primers. The first-round of PCR was performed as follows, 1 cycle of 94°C for 10 min; 35 cycles of 95°C for 45 sec/50°C for 1 min/72°C for 1 min, 72°C for 7 min. The nested PCR was carried out at 1 cycle of 94°C for 10 min; 40 cycles of 95°C for 45 sec/55°C for 2 min/72°C for 2 min; 1 cycle of 72°C for 7 min. PCR products were cloned into the pGEMT-Easy vector (Promega) and transformed into E. coli cells (Novagen, USA) and at least 10 insert positive plasmid clones were sequenced using an ABI PRISM 3730 automated sequencer (Applied Biosystems). The methylation patterns were analyzed in sequences derived from clones with ≥98% cytosine conversions only. All experiments were repeated at least three times for each DMR. The methylation level in each sample was determined by dividing the total number of methylated CpG sites by number of entire CpG sites in ten or more sequenced clones.

### Statistical Analysis

The obtained data of development rates was transformed to arcsine which was then statistically analyzed using Analysis of Variance (ANOVA) along with Duncan's Multiple Range Test (DMRT). All data expressed show mean values ± SEM. A probability of *p*<0.05 was considered to be statistically significant.

## Results

### Developmental potentials of the various types of embryos produced in vitro

The first objective of this study was to evaluate the relative developmental competencies of parthenogenones, androgenones, and biparental fertilized porcine embryos. It is well known that a high incidence of polyspermic penetration occurs during fertilization in the porcine IVF system [24]. Under our experimental conditions, the rate of polyspermy was about 35% (data not shown). In order to eliminate possible contamination with aneuploid embryos, only fertilized oocytes (IVF and AG) showing two pronuclei and parthenogenones with a large pronucleus or two pronuclei were selected. Hoechst 33342 staining at 12 to 14 h after fertilization confirmed the presence of diploid embryos and suitable embryos were then cultured *in vitro*. As shown in Table 2, the cleavage rate of zygotes was noted to be similar amongst IVF (80.4%), parthenogenetic (PG) haploid (74.5%) and diploid (79.7%) embryos. In contrast, only 36.8 and 49.8% of haploid and diploid androgenetic (AG) zygotes underwent cleavage and appeared morphologically normal, the remainder either fragmented rapidly or exhibited delayed and irregular cleavage. Development rate up to the 4-cell stage was not significantly different amongst all types of diploid embryos. However, only 4.9% of AG diploid embryos reached the blastocyst stage, which was significantly lower than IVF and PG diploid embryos (36.1% and 44.3%, respectively, *p*<0.05). Furthermore, the total cell number in AG diploid blastocysts (20.1; n = 10) was significantly lower when compared with IVF and PG diploid blastocysts (78 and 75, respectively, *p*<0.05; n = 10). Amongst the haploid embryos, all AG embryos failed to develop to the blastocyst stage, and only 14.7% of PG embryos developed to the blastocyst stage. Therefore, this study shows that diploid androgenetic embryos produced via the IVF of enucleated oocytes show some success in preimplantation development, but that the overall blastocyst development rate of diploid AG embryos remains inferior to IVF, diploid PG or even haploid PG embryos.

**Table 2:** Developmental potentials of bi and uniparental porcine embryos*.

Method of production	No. zygotes†	No. Cleaved(%)	No. 4 cells(% of cleaved)	No. blastocyst(% of cleaved)	No. cells in blastocyst‡
IVF	583^Diploid^	468 (80.4)^a^	298 (63.6)^a^	170 (36.1)^ab^	78.0^a^
PG	314^Haploid^	234 (74.5)^a^	99 (42.3)^b^	34 (14.7)^b^	38.7^b^
	594^Diploid^	472 (79.7)^a^	317 (67.3)^a^	209 (44.3)^a^	75.7^a^
AG	184^Haploid^	66 (36.8)^c^	14 (22.4)^c^	N/A	N/A
	441^Diploid^	217 (49.8)^b^	133 (61.2)^a^	11 (4.9)^c^	20.1^c^

*The number of replicates was 5.

†Those zygotes having two pronuclei (IVF and AG zygotes) or one large pronucleus or two pronuclei (PG zygotes) were selected after staining with Hoechst 33342.

‡The cells of blastocysts were counted on Day 7.

a–c Values with different letters within each column are significantly different, *p*<0.05.

### Imprinted gene expression patterns in bi- and uniparental diploid embryos from the 2-cell to the blastocyst stage

**Figure 1:**
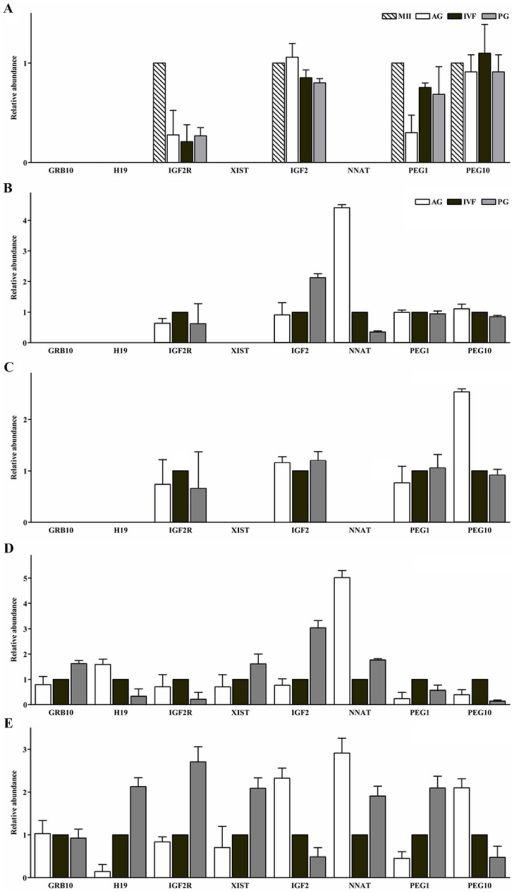
Relative expression levels of the eight imprinted genes in porcine MII oocytes and in diploid normal and uniparental embryos from the two-cell to the blastocyst stage. The relative levels of mRNA were quantified using qRT-PCR and then calculated with the 2^−ΔΔ Ct^ method [23]. Five replicate samples were examined for each class. The values from transcripts of the imprinted genes in PG and AG blastocysts, after normalization relative to the *β ACTIN* (internal control) gene, were compared to those of IVF counterparts which were taken as a standard (1). This data is presented as mean ± SEM. The relative abundance of eight imprinted genes among the different types of embryos at each stage are shown; A; the 2-cell (n = 25), B; the 4-cell (n = 25), C; the 8∼16-cell (n = 15), D; the morula (n = 10), and E; the blastocyst stage (n = 5) of porcine embryos.

To determine the timing of expression of the tested imprinted genes (maternally: *GRB10*, *H19*, *IGF2R*, *XIST*; paternally: *IGF2*, *NNAT*, *PEG1*, *PEG10*) during porcine preimplantation development, we analyzed the mRNA abundance of imprinted genes present in the MII oocyte and at each embryo stage (from two-cell to the blastocyst) amongst three different classes of embryo, IVF, PG and AG diploid embryos respectively (Fig. 1). The cDNA from pooled oocytes or (from the two-cell to the morula) embryos and the individual blastocysts was used for this experiment. Notably, three paternally expressed genes *IGF2*, *PEG1*, *PEG10* and one maternally expressed gene, *IGF2R*, were expressed at detectable levels in most samples at all stages. These expression patterns were stable up to the 16-cell stage but unpredicted or variable expression was seen at the morula stage. Of the genes tested, *GRB10*, *H19*, and *XIST* transcripts were not detected from the oocyte to the 8-cell stage in all classes. However, *GRB10* and *XIST* transcripts started to appear clearly at the 16-cell stage, whereas the *H19* gene was expressed at a detectable level in only a few PG samples. Interestingly, *NNAT* transcripts first appeared at the 4-cell stage but then had disappeared by the 8-cell stage only to reach detectable levels again at the morula stage. In addition, an allele-specific expression pattern was detected for *NNAT* and *PEG10* at 4-cell and morula stages, respectively. Of these, *H19*, *IGF2*, *PEG1*, and *PEG10* were differentially expressed among the classes of blastocyst. *IGF2R* and *XIST* were also more highly expressed in PG blastocysts than in IVF blastocysts with a slightly higher expression of these genes apparent in IVF blastocysts when compared with AG blastocysts. *GRB10* transcripts were nearly equally expressed amongst all classes of embryo. *PEG1* is a maternally imprinted gene but was nonetheless found to be expressed at higher levels in PG blastocysts, relative to AG and IVF counterparts in this experiment. In contrast, the expression level of *NNAT* was much lower in IVF blastocysts than in PG as well as AG blastocysts (Fig. 1E).

### Imprinted gene expression patterns in in vivo and in vitro blastocysts

**Figure 2:**
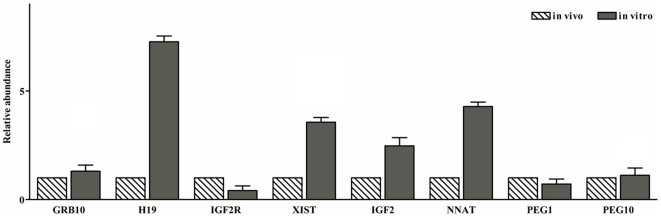
Analysis of imprinted gene expression in in vivo derived and in vitro fertilized blastocysts. Y-value is expressed as a relative fold change in mRNA levels in *in vitro* blastocysts compared with that of the *in vivo* ones defined as 1, (n = 10). This data is presented as mean ± SEM (n = 5).

**Figure 3:**
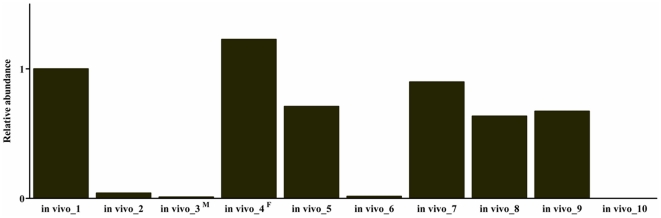
Differential expression of XIST transcripts in individual in vivo blastocysts. Each value derived from transcripts of the *XIST* gene in *in vivo* blastocysts, after normalization relative to *β ACTIN* (internal control), were compared with that of one of 10 *in vivo* blastocysts defined as 1. Of these, the labeled No. 3 and No. 4 samples were determined their sex by SRY gene; ^F^ and ^M^ indicate female and male embryos, respectively.

To further investigate the possible influence of any artefact on a loss of imprinting that may arise from using *in vitro* materials; we extended our study to include in *vivo* blastocysts, as a standard control. Fig. 2 shows that the *H19*, *XIST*, *IGF2* and *NNAT* genes tended to be much more highly expressed in *in vitro* blastocysts than in *in vivo* blastocysts. *IGF2R* genes were transcribed at lower levels in *in vitro* blastocysts than in their *in vivo* counterparts. Furthermore *GRB10*, *PEG1* and *PEG10* genes were all expressed at a similar level in both *in vivo* and *in vitro* blastocysts. These results indicate that the transcriptional activity of several imprinted genes is modulated irregularly in *in vitro* produced embryos. Moreover, we found that the *XIST* transcripts were present in all individual blastocysts derived *in vivo*. In comparing individual *in vivo* blastocysts, it was found that *XIST* transcripts were more highly expressed by at least 100 fold in six out of the ten blastocysts tested (Fig. 3). To account for these individual differences between blastocysts, the sex of embryos representing differential expression patterns (samples labeled No. 3 and No. 4) was identified via the detection of *SRY* gene expression. For this experiment, amplified cDNA was used, as no PCR results could be directly obtained with the initial cDNA concentration in these samples. Clearly, No. 3 for which a low *XIST* expression level was observed *SRY* transcripts were detected by RT-PCR, but no such transcripts were observed in sample No. 4 (data not shown). Taken together, these results indicate that the *XIST* gene is transcribed in both male and female porcine embryos at the blastocyst stage, but the transcriptional activity of the *XIST* gene is regulated differentially under sexual differences within embryos. We must point out that the scope of our analysis did not extend to the *in vitro* embryos. Consequently, although our results show the predicted expression pattern between different types of blastocysts, this may be complicated by no distinction based upon sex in them, especially in IVF and AG embryos.

### Relationship between allele expression and ploidy in parthenogenetic blastocysts

**Figure 4:**
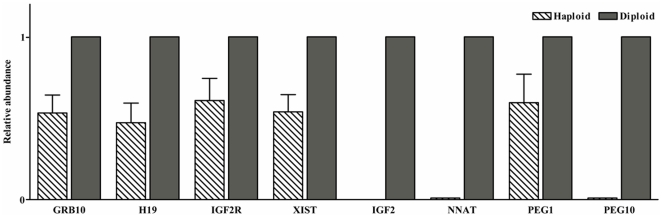
Imprinted gene expression of haploid and diploid PG blastocysts. Haploid PG blastocysts were generated using the electrical activation method without cytochalasin D treatment. Zygotes possessing two polar bodies and a small pronucleus (presumed haploid) or with a polar body and a large pronucleus or two pronuclei (presumed diploid) were selected by Hoechst staining at 12 to 14 hr following parthenogenetic activation, respectively. Results for each sample were conducted in triplicate. Y-value is expressed as a relative fold change in mRNA levels in haploid PG blastocysts (n = 5) compared with that of the diploid ones (n = 5) defined as 1. The Data are presented as means ± SEM.

**Figure 5:**
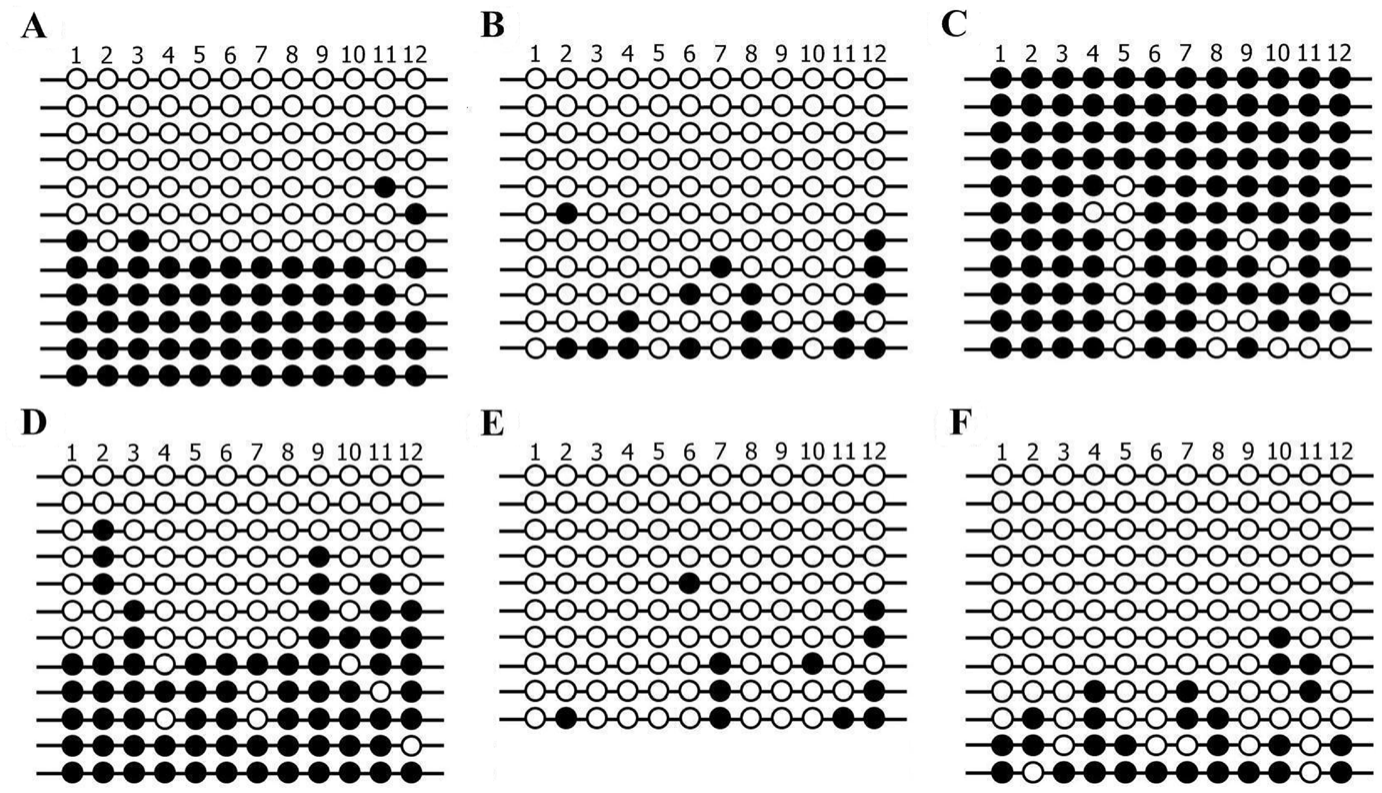
The methylation status of *IGF2/H19* DMR3 in porcine haploid and diploid PG blastocysts. The methylation patterns of DMR3 in porcine A; adult liver tissue (1×10^5^ cells), B; MII oocytes (n = 100), C; sperm (1×10^6^ sperm cells), D; IVF (n = 50), E; haploid PG (n = 100), and F; diploid PG (n = 50) blastocysts are shown. Individual circles indicate a CpG dinucleotide. Open and solid circles represent unmethylated and methylated CpGs, respectively. Each horizontal line represents one individual clone from three independently amplified PCR products.

Interestingly, all genes tested were expressed in both uniparental embryos, but maternally and paternally expressed genes should not be expressed in androgenones and parthenogenones, respectively. Indeed, this phenomenon has previously been recorded in studies involving uniparental mouse embryos, and has been linked to dosage compensation in diploid cells [25]. We were therefore interested to investigate a possible relationship between imprint expression and ploidy in porcine embryos. To gain insight into this phenomenon, the expression pattern and methylation status of diploid and haploid PG blastocysts was examined. Unfortunately, as no haploid AG embryos developed into blastocysts, it was not possible to consider AG embryos in this experiment. As shown in Fig. 4, *IGF2*, *NNAT* and *PEG10* did not display detectable levels of expression in haploid PG blastocysts while *GRB10*, *H19*, *IGF2R*, *XIST* and *PEG1* were expressed at lower levels in haploid PG blastocysts in comparison to their diploid counterparts. These results indicate that the paternally expressed genes, with the exception of *PEG1*, were activated in diploid PG blastocysts, but not in haploid PG blastocysts. The methylation status of *IGF2/H19* DMR3 in haploid and diploid PG blastocysts was investigated using the bisulfite genomic sequencing assay. The results presented in Fig. 5A–C show that these regions in MII oocytes were unmethylated (13.6%) and most of the CpGs in sperm methylated (78.1%), whilst a hemimethylation pattern (43.1%) was seen in adult liver tissue. The results also showed that several sequenced clones were heavily methylated in diploid PG embryos (18.7%) (Fig. 5F), whereas that this region in haploid PG blastocysts remains unmethylated with the partly methylated CpG sites in several sequenced clones (9.1%) (Fig. 5E). Moreover, the observed methylation pattern in diploid PG blastocysts was different from that of the IVF blastocysts (40.9%) (Fig. 5D), indicating that the methylation status of this region in diploid PG blastocysts was partially altered.

## Discussion

### Developmental potential of bi- and uniparental embryos

Parthenogenetic embryos can be easily generated by oocyte activation via a variety of treatments such as a brief exposure of Ca^++^ or ethanol and electrical activation [26]. Androgenetic embryos have been generated by pronuclear transfer (PT); physically transferring pronuclei between zygotes, and to date this has been the most widely used method for producing mouse androgenones [2]. However, this PT method requires great effort with skilled manipulations and is indeed impracticable for some species where there is no significant difference in size between the two pronuclei. Furthermore this method often requires an additional procedure in order to visualize the pronuclei within a cytoplasm containing opaque lipids [27]. An alternative method utilizing the fertilization of enucleated oocytes, first reported by Kono *et al.* [22], has recently been applied to some species. It has been suggested that this method would be suitable for producing bovine androgenones, where embryos derived via ICSI of enucleated oocytes display a retarded development at early cleavage stages [27]. Here we have shown that porcine diploid androgenones produced by the IVF of enucleated oocytes are developmentally competent up to the blastocyst stage.

### Imprinted gene expression pattern in bi- and uniparental embryos

The results presented here show that the expression levels and patterns recorded for most imprinted genes in oocyte samples, except for *NNAT*, up to the 16-cell stage of development, occurred regardless of either the embryos parental-origin or the direction of imprinting. At the morula stage, all tested genes were highly expressed in all classes, whereas the expression levels fluctuated from embryo to embryo. Consequently, these genes appeared to be transcriptionally active in an inconsistent manner at this stage with no apparent monoallelic expression at the morula stage. Of the maternally expressed genes, *GRB10* and *H19* were transcriptionally repressed in the majority of embryos until the 4-cell stage. These transcripts were then detected in some embryos as early as the 8-cell stage but were detected in all by the morula stage. At the blastocyst stage, *H19* exhibited a parental specific expression pattern among the different classes. A previous study has demonstrated a monoallelic expression pattern in mouse pre- and postimplantation embryos for the *H19* gene [28]. Furthermore, our preliminary experiments have found that the methylation imprint of this gene is established through porcine preimplantation development [11]. In case of *GRB10*, it has been found to be expressed in human blastocysts, but as yet no evidence has been provided for an allele-specific expression [29]. A recent study has shown that *GRB10* is expressed biallelically in ovine blastocysts [30], which is consistent with our data, as this expression level appeared to be almost indistinguishable amongst the different classes. Our findings show that *XIST* transcripts were detectable in all blastocysts, although these expression levels in both individual IVF and AG samples were variable with regards to other imprinted genes tested. This result indicates that the *XIST* gene in both maternal X (X^M^) and paternal X (X^P^) chromosomes in porcine embryos are expressed. This is consistent with results from human embryos that show that the transcripts are revealed in both male and female blastocysts [31]. However, the mouse *Xist* gene shows a preferential expression in X^P^ in the extraembryonic lineage, consequently, only X^M^ is generally active in blastocysts [19]. It has become evident that the essential sequences for imprinted *Xist* expression in mice are not conserved in humans [32]. Interestingly, our results also show that whilst there was a distinct difference between male and female blastocysts, *XIST* transcripts were clearly expressed in both *in vivo* male and female blastocysts. The expression of *XIST* was indeed much higher in female blastocysts, indicating that imprinted *XIST* expression in pigs is involved in the regulation of XCI. These findings suggest that imprinted *XIST* expression occurs in preimplantation stages, since *XIST* transcripts from X^M^ were preferentially expressed in *in vivo* porcine blastocysts. A distinct expression pattern of the *NNAT* gene has been detected in bovine embryos; *NNAT* is expressed until the 4-cell stage, repressed by the 8-cell stage, but then reappears at the blastocyst stage [33]. This expression pattern is similar to our observed expression patterns. It has been proposed that some imprinted gene activation occurs following maternal-to-zygotic transition (MZT) [34]. MZT varies across different species; it occurs during the late 2-cell stage in the mouse, while it occurs at the 4-cell stage in pigs, and the 8- to 16-cell stage in bovine and ovine embryos [35]. With this in mind, it may be thought that the porcine *NNAT* gene is likely to follow this trend. However, an apparent differential expression was not found among all classes but was recorded between IVF and AG blastocysts. Our results show that the *IGF2* and *IGF2R* transcripts were detected in porcine oocytes and all classes throughout preimplantation development. *IGF2* revealed a differential expression with a 2-fold increase or decrease among the different types of blastocysts. Similar results have also demonstrated that the transcripts of human *IGF2* and *IGF2R* are found to be expressed throughout preimplantation development as well as imprinted from the 8-cell stage onwards [36]. This is consistent with the results from uniparental mouse embryos suggesting that diploid PG embryos express a low level of *Igf2* mRNA in comparison to that of AG embryos [34]. A three-fold increase in *IGF2R* expression was found in PG blastocysts when compared with IVF and AG counterparts, but no difference was recorded between IVF and AG blastocysts. This is comparable with previous reports showing a higher *Igf2r* expression in PG fetuses compared with that of control mouse fetuses [37]. Our results show that the *PEG1* and *PEG10* transcripts were detected in porcine oocytes and all embryo types from the 2-cell to the blastocyst stage. *PEG1* displays a parental-specific expression but in an opposite direction at the blastocyst stage. This is in contrast with the previous observation of methylation imprints established in early mouse embryos [15]. It has been suggested that some genes exhibit discrepant imprinting differences between species as well as different tissues [38]. In the case of *PEG10* gene, an apparent differential expression was discovered amongst the different classes at the morula and blastocyst stage. Although, comparable data for the allelic status of the *PEG10* gene in preimplantation embryos is still insufficient for most species, it has been suggested that the human *PEG10* gene exhibits a paternal expression pattern at the blastocyst stage [39]. These results demonstrate that each gene has its own time window to receive primary imprinting during early pig development and imprinted expression in porcine blastocysts occurs in at least four genes, namely *H19*, *IGF2*, *XIST* and *PEG10*.

### Imprinted gene expression patterns in in vivo and in vitro blastocysts

This study addressed questions regarding possible influences on the loss of imprinting that may arise from the use of *in vitro* materials, such as those used in *in vitro* culture and techniques used in manipulations, such issues have been inferred from previous studies in mice and humans [4]. We also found that the expression of some genes was altered in *in vitro* blastocysts from expression levels found in their *in vivo* counterparts. Previous studies showed that the *H19* gene is highly susceptible to *in vitro* conditions [5], [40]. This is comparable with our result showing higher *H19* expression in *in vitro* blastocysts compared with that of *in vivo* blastocysts. The partial methylation pattern in *IGF2*/*H19* DMR of *in vitro* materials may be caused by environmental conditions, as reported previously [11]. However, the observed disruptions in methylation were less dramatic in *in vitro* blastocysts, considering their *H19* expression pattern. Although the discrepancy between *H19* expression and methylation remains unclear, it is possible that imprinted expression of *H19* is affected by *in vitro* conditions whilst appropriate allele-specific methylation at the DMR occurs in *in vitro* blastocysts. The *NNAT* gene was transcribed in *in vitro* blastocysts at a level fourfold higher than that of *in vivo* blastocysts. This is in line with the previous microarray studies of altered imprint expression statuses established in *in vitro* porcine preimplantation embryos [41]. These findings appear to indicate that *in vitro* culture conditions may result in the aberrant expression of some imprinted genes in resulting blastocysts. However, this phenomenon is difficult to evaluate conclusively, and remains largely undefined in the porcine species. Further studies are therefore required in order to fully evaluate the effects of various experimental conditions.

### Relationship between imprint expression and ploidy

Although our data showed a two-fold difference in mRNA abundance according to parental origins for most genes among the classes, inappropriate expression for some genes was also observed in uniparental embryos. Interestingly, this may well be because it has been found that parental specific expression occurs in PG and AG embryos without the participation of both parental genomes. It has been shown that this phenomenon takes place in diploid uniparental embryos but not in haploid [25]. It has also been proposed that imprints in uniparental mouse embryos, for some genes, can be appropriately adjusted by dosage compensation or counting mechanisms [34]. Our results confirmed that the abundant expression of *IGF2*, *NNAT* and *PEG10* as seen in diploid PG blastocysts was either transcriptionally silenced or expressed weakly in haploid PG blastocysts. Furthermore, this study consistently showed that the degree of methylation in diploid PG blastocysts was higher than that seen in haploid PG blastocysts which have the similar imprinted pattern as mature oocytes, indicating that disruptions may be not solely responsible for *in vitro* culture. These findings suggest that the appropriate expression of several paternally expressed genes occurs even in diploid PG embryos, but not in haploid counterparts. This phenomenon may be associated with an altered methylation status at an imprinting control region. However, it also implies that complete imprinting can be achieved only within embryos consisting of both parental alleles.

Primarily we have shown here, by comparing mRNA expression levels in bi- and uniparental embryos, the imprinted expression status of imprinted genes in the preimplantation porcine embryo. Several previous studies have accounted for unregulated gene expression in uniparental embryos by suggesting that it appears as a result of gene dosage compensation in diploid cells or via a loss of imprinting [25], [37]. This phenomenon was also recorded in this study. Therefore it is necessary to consider the possibility of misinterpretation when imprinted gene expression data is derived from diploid uniparental embryos. Given this consideration, the comprehensive analysis of combined sets of data, considering ploidy in uniparental embryos, may be necessary to provide a more robust means of measuring imprinted gene expression during preimplantation development.
